# Kupffer Phase Radiomics Signature in Sonazoid Contrast‐Enhanced Ultrasound Predicts Immunohistochemistry Marker Expression in Hepatocellular Carcinoma

**DOI:** 10.1002/cam4.71153

**Published:** 2025-10-06

**Authors:** Chen Li, Yuan Liu, Mingxiao Wu, Weide Dai, Jinghai Song, Yong Wang

**Affiliations:** ^1^ Department of Ultrasound, National Cancer Center/National Clinical Research Center for Cancer/Cancer Hospital Chinese Academy of Medical Sciences and Peking Union Medical College Beijing China; ^2^ Department of Ultrasound, Beijing Hospital, National Center of Gerontology, Institute of Geriatric Medicine Chinese Academy of Medical Sciences & Peking Union Medical College Beijing China; ^3^ Department of General Surgey, Beijing hospital, National Center of Geronotology, Institute of Geriatric Medicine Chinese Academy of Medical Sciences & Peking Union Medical College Beijing China; ^4^ Department of Ultrasound The first Affliated Hospital of China Medical University Shenyang China

**Keywords:** contrast‐enhanced ultrasound, hepatocellular carcinoma, immunohistochemical markers, radiomics

## Abstract

**Purpose:**

Few studies have explored the value of radiomics signatures in predicting immunohistochemical (IHC) staining markers. This study aimed to investigate and validate radiomics models based on the Kupffer phase of Sonazoid contrast‐enhanced intraoperative ultrasonography (S‐CEUS) images for predicting IHC marker expression in hepatocellular carcinoma (HCC).

**Method:**

Overall, 113 consecutive patients diagnosed with HCC between November 2019 and May 2023 were retrospectively analyzed. Histopathological assessment included IHC staining for GS, CD10, GPC3, and HSP70. Radiomic features extracted from S‐CEUS images were selected and analyzed. A Naïve Bayes classifier was employed to predict IHC marker expression in HCC, using selected clinical biomarkers and radiomic features.

**Results:**

For GPC3, the radiomics classifier achieved a macro‐average area under the receiver operating characteristic curve (AUC) of 0.700, indicating strong performance. For GS, both radiomics and combined clinical‐radiomics classifiers exhibited strong discrimination (AUCs: 0.870 and 0.882, respectively). The radiomics classifier outperformed clinical biomarkers (total and direct bilirubin) in predicting CD10, with a macro‐average AUC of 0.834. However, its accuracy decreased for higher HSP70 marker expression levels (AUC: 0.694). These findings underscore the consistent effectiveness of radiomics across different IHC markers when compared to traditional clinical approaches.

**Conclusions:**

The Kupffer phase in the S‐CEUS‐based radiomics signature is an excellent biomarker for predicting IHC marker expression in patients with HCC.

**Abbreviations**
ALT: alanine transaminase
ANOVA: analysis of variance
ANOVA‐Spearman: Spearman correlation of ANOVA
AST: aspartate transaminase
AUC: area under the ROC
CA125: carbohydrate antigen 12‐5
CA153: carbohydrate antigen 15‐3
CA199: carbohydrate antigen 19‐9
DBil: direct bilirubin
E‐S: Edmondson–Steiner
GPC3: glypican‐3
GS: glutamine synthetase
HCC: hepatocellular carcinoma
HSP70: heat shock protein 70
IHC: immunohistochemical
NRI: net reclassification improvement
ROC: receiver operating characteristic
S‐CEUS: Sonazoid contrast‐enhanced ultrasound
TBil: total bilirubin

## 1 Introduction

Hepatocellular carcinoma (HCC), one of the most common primary hepatic malignant tumors, is the third leading cause of cancer‐related deaths worldwide, with a 5‐year survival rate of < 20% [1, 2]. Several therapies, such as hepatic surgical resection, transplantation, and transcatheter arterial chemoembolization, are effective and commonly used to treat HCC. Among these, hepatectomy is the preferred therapeutic method for most patients with HCC. The prognosis of HCC has improved with the advances in hepatectomy and imaging technologies. However, its high potential for vascular invasion, metastasis, and recurrence post‐resection leads to a poor prognosis [3, 4].

Glypican‐3 (GPC3), glutamine synthetase (GS), and heat shock protein 70 (HSP70) are biomarkers currently used to discriminate the nature of hepatocellular lesions smaller than 2 cm detected in patients with liver cirrhosis, which lack the radiological features of HCC [5]. CD10 is a tissue marker used to confirm the diagnosis of HCC through imaging in histology [5]. Among the prognostic factors for HCC, GPC3 has been closely associated with postoperative metastasis/recurrence in patients with HCC [6]. The expression of GPC3 in HCC is an important independent factor in predicting a patient's poor prognosis. International guidelines recommend the combined use of these immunohistochemical (IHC) markers for a more accurate diagnosis. Compared with early HCC, assessable using this panel of markers, our clinical challenge involved distinguishing dysplastic nodules [7].

Radiomics analysis is an emerging medical imaging technique that has attracted considerable attention in recent years. Radiomics can be used to extract high‐dimensional information that is invisible to humans [8]. This feature could be used in the characterization of tumor heterogeneity, as well as reflecting the tumor tissue microenvironment and cancer phenotype [9]. Consequently, the radiomics signature has become a prognostic biomarker that can augment available clinical data, aid in lesion detection, improve the accuracy of diagnosis, predict the risk of disease, and determine treatment strategies [10]. Ultrasound, particularly Sonazoid contrast‐enhanced ultrasound (S‐CEUS), has been successfully applied to predict the pathological grading of tumors, evaluate malignancy, and assess treatment response with liver‐specific contrast agents such as Sonazoid. This agent enables prolonged Kupffer phase imaging, more effectively characterizing focal liver lesions [11, 12]. Several studies have reported that radiomics signatures derived from S‐CEUS show potential in predicting microvascular invasion, histopathological grade, and Ki‐67 protein expression levels in HCC [12, 13, 14, 15]. However, to our knowledge, few studies have attempted to identify the potential of radiomics signatures in predicting other IHC markers, such as GPC3, GS, HSP70, and CD10. This study aimed to investigate and validate the performance of radiomics models based on the Kupffer phase of S‐CEUS images in predicting the expression of IHC markers in HCC.

## 2 Materials and Methods

### 2.1 Patients

Ethical approval was obtained from the Institutional Review Board (2021BJYYEC‐190‐02), which waived the requirement for informed consent. All procedures were performed in accordance with the Declaration of Helsinki.

A total of 113 consecutive patients with HCC were retrospectively considered between November 2019 and May 2023. The inclusion criteria were as follows: (1) patients with a suspected diagnosis of HCC; (2) possession of complete medical information; (3) having undergone preoperative grayscale ultrasound and Kupffer phase imaging of S‐CEUS; (4) no previous treatments, such as radiofrequency ablation, microwave ablation, and chemotherapy; and (5) a pathologic report confirming HCC, with routine immunochemical staining for GS, CD10, and GPC3 conducted. The exclusion criteria were: (1) absence of S‐CEUS; (2) incomplete clinical information; (3) lack of an available IHC report; (4) contraindication for the use of the Sonazoid contrast agent; and (5) poor B‐mode or S‐CEUS image quality of focal liver lesions.

Potential clinical biomarkers were identified through patient interviews and a thorough review of medical records. The clinical characteristics of the patients included age, sex, maximum tumor diameter, alpha‐fetoprotein levels, total bilirubin (TBil), direct bilirubin (DBil), carbohydrate antigen 12‐5 (CA125), carbohydrate antigen 19‐9 (CA199), carbohydrate antigen 15‐3 (CA153), aspartate transaminase (AST), and alanine transaminase. Data on risk factors, including hepatitis and alcohol consumption, were collected and recorded.

### 2.2 Histopathological Result and Immunohistochemical Staining Procedure

Histopathological results, including the Edmondson–Steiner (E‐S) grade and IHC markers, were evaluated by a pathologist with > 10 years' experience. The resected specimens were fixed in 10% paraformaldehyde, embedded in paraffin, and cut into 4‐μm‐thick sections for hematoxylin–eosin staining or IHC identification. The histological grade of HCC was determined based on the E‐S grade [16]. Grades I and II were classified as low grade, whereas grades III and IV were classified as high grade. For the IHC analysis, the expression levels of GS, CD10, and GPC3 were categorized. Negative expression (no immunoreactivity, −), mildly positive expression (< 50% immunoreactivity, +), and strongly positive expression (> 50% immunoreactivity, ++) were identified based on the staining patterns [16, 17].

### 2.3 Sonazoid Contrast‐Enhanced Ultrasound Imaging

All patients underwent routine grayscale ultrasonography and S‐CEUS. Two sonographers conducted the S‐CEUS examinations using an Aplio 500 (Canon), equipped with convex (6C1, 1–6 MHz) and linear (11L4, 4–11 MHz) probes, and an Aplio i800 (Canon), equipped with convex (PVI‐475BX, 1–8 MHz) and linear (11L4, 4–11 MHz) probes. According to the size of the lesion, the mechanical index of the acoustic output was set to 0.19–0.22 with a dynamic range of 65–70 dB. The patients were injected with 0.5 mL of Sonazoid through a peripheral venous line, followed by 5 mL of saline. The enhancement features were recorded and analyzed according to the latest WFUMB guidelines [11]. The Kupffer phase was obtained by scanning for 15 min. The best frames were identified from the grayscale ultrasound image, and the Kupffer phase image was sampled for radiomic analysis.

### 2.4 Image Processing and Radiomics Feature Acquisition

**FIGURE 1:**
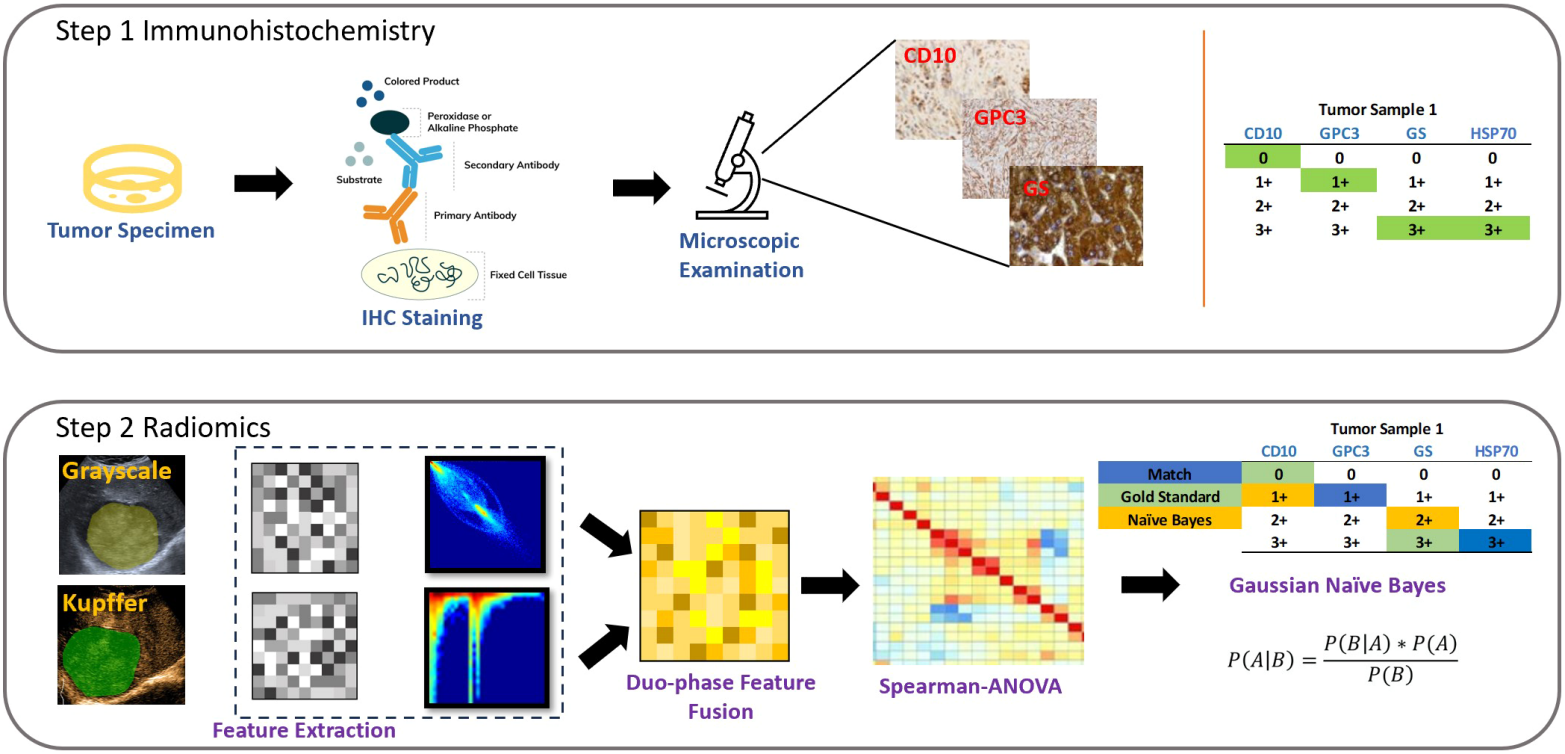
Overview of the study workflow. The process is divided into two main steps. Step 1: Immunohistochemistry (IHC) involves preparing tumor sections, performing IHC staining, microscopic examination, and evalating the expression status of markers CD10, GPC3, GS, and HSP70. Step 2: Radiomics analysis includes lesion annotation on grayscale and Kupffer phase contrast‐enhanced ultrasound images, radiomic feature extraction, biphasic feature fusion, feature selection using Spearman correlation and ANOVA, and model construction with Naïve Bayes classification.

Tumor region of interests (ROIs) were manually delineated by two experienced radiologists (each with over 10 years of ultrasound imaging expertise) using ITK‐SNAP software (version 3.8.0). The segmentation criteria included the entire tumor volume visible on BM and KP images, excluding necrotic areas and major blood vessels identified by hypoechoic or hyperechoic regions. Contours were drawn on 2D slice of the tumor's maximum cross‐sectional area. Radiomic features were extracted using NovoUltrasound Kit (NUK V1.5.0, GE HealthCare). A total of 107 features were computed per ROI, including first‐order statistics (e.g., mean, median, 90th percentile), gray‐level co‐occurrence matrix (GLCM, e.g., cluster shade, inverse variance), gray‐level run length matrix (GLRLM, e.g., run entropy), gray‐level size zone matrix (GLSZM, e.g., zone entropy), gray‐level dependence matrix (GLDM, e.g., dependence variance), and neighborhood gray‐tone difference matrix (NGTDM, e.g., busyness). Features were extracted from the original images and after applying the wavelet, LoG, and LBP filters, resulting in 1284 features per ROI (107 features × 12 filter combinations). To address potential batch effects from dual ultrasound systems (Aplio 500/i800), ComBat harmonization was applied to normalize radiomic features across systems. The feature values were normalized based on the *Z*‐score. Radiomic features from B‐mode, arterial phase, and Kupffer phase ultrasound images were fused into a single feature vector for analysis (Figure 1).

To assess feature stability against interobserver variability in ROI delineation, a subset of 30 nodules was independently segmented by a third radiologist (8 years of experience). Intraclass correlation coefficients (ICCs) were calculated for the selected radiomic features using a two‐way random‐effects model (absolute agreement, single rater). Features with an ICC > 0.8 were retained for analysis.

### 2.5 Multiclass Prediction of IHC Markers

To address the high dimensionality and potential redundancy among radiomic features, we implemented a comprehensive feature selection process that combined analysis of variance (ANOVA) and Spearman's rank correlation coefficient (ANOVA‐Spearman). The ANOVA‐Spearman pipeline retained features with ANOVA *p* < 0.05 to ensure significant variance across IHC groups and removed features with Spearman's *ρ* > 0.75 to reduce redundancy. For predictive modeling, we adopted a Naïve Bayes classifier, a well‐established method in multiclass classification tasks, to classify the IHC markers effectively. A grid search was performed over the Naïve Bayes classifier's bandwidth values ranging from 0.01 to 1.0 (in increments of 0.01) to maximize the AUC on the training data within each leave‐one‐out cross validation (LOOCV) fold. To reduce overfitting during tuning, the bandwidth range was constrained to avoid excessively narrow distributions that could fit noise in the training data. Two separate models were developed: the first utilized ANOVA‐selected clinical biomarkers, while the second integrated both clinical biomarkers and radiomic features. To ensure robust model validation, we employed the LOOCV technique, which evaluates the model's performance by iteratively training it on all but one sample and testing it on the excluded sample. This method was chosen to mitigate overfitting and provide a more generalized performance estimate. The combined model, incorporating both clinical biomarkers and radiomic features, is expected to leverage complementary information, potentially enhancing the predictive accuracy for IHC marker classification (Figure 1).

### 2.6 Statistical Methods

Descriptive statistics for continuous clinical biomarkers are presented either as mean ± standard deviation or median (interquartile range), depending on their statistical distribution determined by the Shapiro–Wilk test (*p* value > 0.05 indicates normal distribution). The 95% confidence intervals were determined via bootstrapping (1000 resamples). Accuracy was used to provide a straightforward measure of the proportion of correct predictions. The receiver operating characteristic (ROC) and the area under the ROC (AUC) curve were utilized to assess the discriminative power of individual prediction classes. The F1 score denoted the harmonic mean of precision and recall. The macro‐averaged AUC offered an aggregate perspective on discrimination performance across all classes. Cohen's kappa measures chance correlations on a scale from 0 to 1, where 1 indicates optimal reliability, and values below 0 indicate performance inferior to randomness. It is categorized as slight, fair, moderate, substantial, or perfect [18]. The net reclassification improvement (NRI) metric was used to quantitatively evaluate the combined model's proficiency in accurately reclassifying subjects compared with a benchmark model, specifically the clinical biomarker model in this context. An NRI exceeding 0 indicates enhanced classification. To address potential intra‐patient lesion correlation in patients with multiple nodules (*n* = 20), a supplementary mixed‐effects logistic regression model was applied, with patient ID as a random effect to account for clustering. *p* values < 0.05 denoted statistical significance. SHapley Additive exPlanations (SHAP) analysis was performed to quantify the contribution of radiomic features to the Naïve Bayes (NB) classifiers for GS, CD10, GPC3, and HSP70 expression.

## 3 Results

### 3.1 Patient Characteristics and IHC Marker Distribution

A total of 86 patients with 106 histologically confirmed HCC nodules were enrolled in this study, with 89.6% male and an average age of 62.02 ± 10.21 years. The distribution of IHC markers across these nodules was as follows: 7 nodules (6.6%) were GS negative, 40 nodules (37.7%) were GS mildly positive, and 59 nodules (55.7%) were GS strongly positive. Regarding CD10 expression, 31 nodules (29.2%) were negative, 67 nodules (63.2%) were mildly positive, and 8 nodules (7.5%) were strongly positive. Additionally, significant differences in pathological grade were observed across the classes of GS and GPC3 expression. TBil and DBil were identified as significant clinical biomarkers for CD10 expression (Table 1).

**TABLE 1:** Baseline characteristics of patients and test results for different IHC classes.

Biomarkers	Population (*N* = 106)	ANOVA/K‐W (GPC3)		ANOVA/K‐W (CD10)		ANOVA/K‐W (GS)		ANOVA/K‐W (HSP70)	
		Statistics	*p*	Statistics	*p*	Statistics	*p*	Statistics	*p*
Sex		0.851	0.430	0.991	0.374	0.263	0.769	1.459	0.220
Female	11 (10.4)								
Male	95 (89.6)								
Age (years)	62.02 ± 10.21	0.324	0.724	2.922	0.058	0.806	0.450	1.453	0.231
Tumor size (mm)	3.30 [2.00, 5.00]	0.046	0.955	0.365	0.695	1.677	0.192	1.833	0.145
AFP	6.8 [3.30, 155.78]	1.917	0.152	0.359	0.699	1.469	0.234	1.286	0.283
TBIL	15.20 [10.00, 21.73]	0.813	0.446	7.749	< 0.001	0.749	0.475	0.028	0.993
DBIL	5.00 [3.60, 8.5]	1.011	0.367	7.363	0.001	0.679	0.509	0.164	0.920
AST	30.00 [21.00, 51.75]	0.540	0.584	0.455	0.635	0.941	0.394	0.132	0.339
ALT	26.00 [17.00, 52.75]	0.825	0.441	0.499	0.608	1.123	0.329	0.294	0.829
CA125	14.95 [10.20, 30.23]	1.176	0.312	1.339	0.267	0.046	0.955	1.110	0.347
CA199	9.45 [4.62, 19.77]	0.577	0.563	0.821	0.443	1.372	0.285	0.216	0.884
CA153	9.10 [6.72, 11.97]	0.109	0.896	0.004	0.995	2.874	0.061	1.771	0.157
Risk factor		1.781	0.174	1.898	0.155	0.402	0.672	1.979	0.122
Hepatitis	92 (86.8)								
Alcohol use	13 (12.3)								
Other	1 (0.9)								
Pathological grade		6.136	0.003	2.603	0.079	4.048	0.020	1.299	0.278
Low	59 (55.7)								
High	47 (44.3)								

*Note:* Descriptive statistics are reported as the mean ± standard deviation or median [interquartile range]. *p* values < 0.05 for the ANOVA indicate statistically significant difference between IHC classes.

Abbreviations: AFP, alpha‐fetoprotein; ALT, alanine aminotransferase; ANOVA, analysis of variance; AST, aspartate aminotransferase; DBIL, direct bilrubin; GPC3, glypican‐3; GS, glutamine synthetase; HSP70, heat shock protein 70; IHC, immunohistochemical; TBIL, total bilrubin.

### 3.2 Prediction Results for IHC Marker GS

**FIGURE 2:**
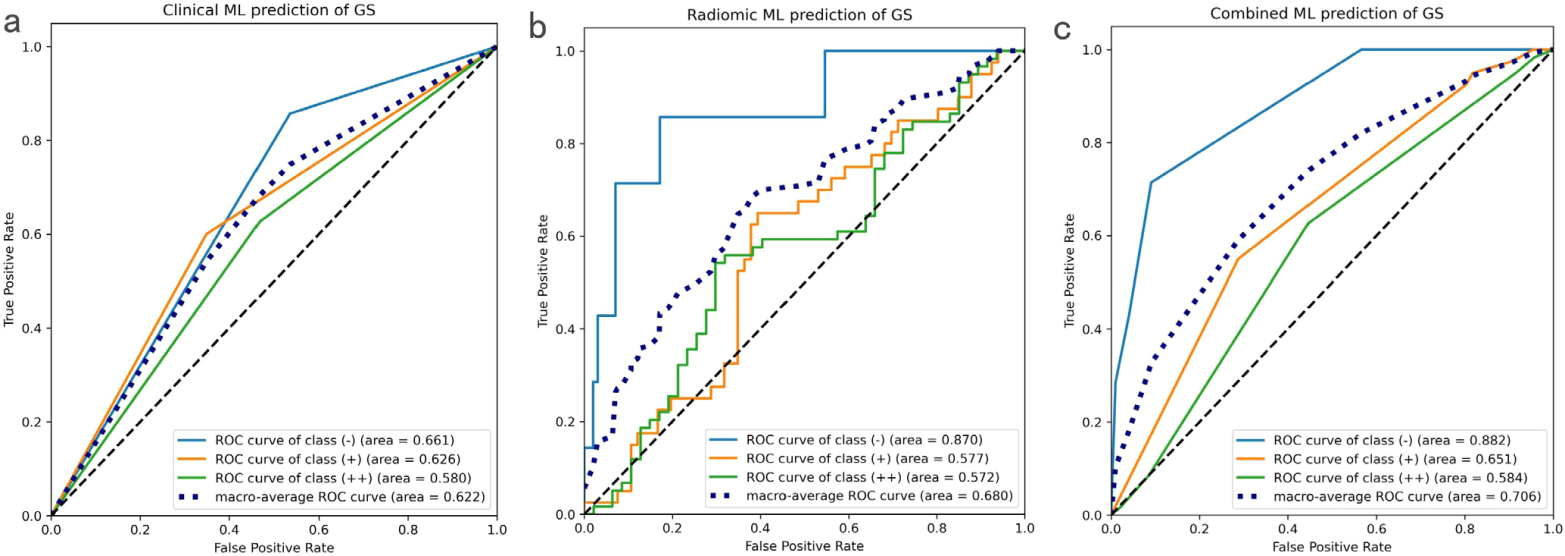
ROC curves for clinical, radiomics, and combined machine learning prediction of GS. ROC curves for the clinical (pathological grade) classifier (a), radiomics classifier (b), and the combined clinical‐radiomics classifier (c) for the multiclass prediction of GS. The combined classifier achieved an AUC of 0.882, outperforming the clinical classifier (AUC = 0.661) and radiomics classifier (AUC = 0.870). The macro‐averaged AUC of the combined classifier was 0.706.

In the analysis for GS expression, a total of 11 radiomic features from the B‐mode ultrasound and 12 features from the Kupffer phase of S‐CEUS were identified as relevant by the ANOVA–Spearman feature selection process (Table 2) (Table S1, Table S5). The ROC curves for classifiers based on clinical data, radiomics, and a combined clinical‐radiomics approach are presented in Figure 2. The SHAP summary bar plot (Figure S1) ranks the mean absolute SHAP values for all 23 features. The top‐ranking features included log‐sigma‐0‐2‐mm‐3D_firstorder_90Percentile (BM, SHAP value = 0.374), original_gldm_DependenceVariance (BM, SHAP value = 0.341), and lbp‐2D_firstorder_Energy (KP, SHAP value = 0.301). Lower‐ranking features included wavelet‐LH_glszm_LargeAreaHighGrayLevelEmphasis (KP, SHAP value = 0.054). The combined Naïve Bayes classifier, integrating both clinical biomarkers and radiomic features, demonstrated superior AUC values compared to the classifiers using clinical or radiomic data alone. Specifically, the combined classifier achieved an AUC of 0.882, outperforming the clinical classifier (AUC = 0.661) and radiomics classifier (AUC = 0.870). The macro‐averaged AUC, reflecting overall performance across all classes, was 0.706. These results indicate that the combined method was more effective in classifying GS expression than the clinical method alone. However, the addition of pathological grades to the radiomics analysis did not further improve these results.

**TABLE 2:** ANOVA‐Spearman selected radiomics features for GS expression classification.

B‐mode	Kupffer
log‐sigma‐0‐2‐mm‐3D_firstorder_90Percentile	lbp‐2D_firstorder_Energy
log‐sigma‐0‐3‐mm‐3D_ngtdm_Strength	log‐sigma‐0‐1‐mm‐3D_firstorder_Mean
log‐sigma‐0‐4‐mm‐3D_firstorder_Uniformity	log‐sigma‐0‐2‐mm‐3D_firstorder_TotalEnergy
original_glcm_DifferenceAverage	log‐sigma‐0‐2‐mm‐3D_glcm_ClusterShade
original_gldm_DependenceVariance	log‐sigma‐0‐2‐mm‐3D_glcm_Contrast
wavelet‐HL_firstorder_Median	log‐sigma‐0‐3‐mm‐3D_glszm_SmallAreaLowGrayLevelEmphasis
wavelet‐HL_glcm_Imc1	original_firstorder_MeanAbsoluteDeviation
wavelet‐HL_glcm_Imc2	original_glcm_ClusterShade
wavelet‐HL_glrlm_RunEntropy	original_glcm_InverseVariance
wavelet‐HH_glcm_Correlation	original_glcm_JointEnergy
wavelet‐HH_glszm_SizeZoneNonUniformityNormalized	original_glszm_LargeAreaHighGrayLevelEmphasis
	wavelet‐LH_glszm_LargeAreaHighGrayLevelEmphasis

Abbreviations: ANOVA, analysis of variance; GS, glutamine synthetase.

In terms of diagnostic performance, the combined classifier achieved the highest accuracy, F1 score, and Cohen's kappa (Table 3). Additionally, both the radiomics and combined methods exhibited a positive NRI compared with the clinical method, with NRI values of 0.367 and 0.454, respectively. A positive NRI of 0.048 was also observed when comparing the combined method to the radiomics classifier, suggesting that the addition of radiomic features improved the classification results of the clinical biomarkers.

**TABLE 3:** Diagnostic summary for GS classifiers.

	Clinical classifier			Radiomic classifier			Joint classifier		
	(−)	(+)	(++)	(−)	(+)	(++)	(−)	(+)	(++)
(−)	0	1	6	3	2	2	3	0	4
(+)	0	24	16	1	2	37	1	22	17
(++)	0	22	37	3	5	51	1	19	37
Accuracy	0.576 (95% CI: 0.572, 0.581)			0.529 (95% CI: 0.525, 0.533)			0.588 (95% CI: 0.584, 0.592)		
F1 score	0.393 (95% CI: 0.390, 0.397)			0.383 (95% CI: 0.378, 0.389)			0.514 (95% CI: 0.508, 0.520)		
Cohen's *κ*	0.192 (95% CI: 0.184, 0.199)			0.044 (95% CI: 0.038, 0.050)			0.243 (95% CI: 0.236, 0.250)		
Per‐class AUC	0.661	0.626	0.580	0.870	0.577	0.572	0.882	0.651	0.584
Macro‐averaged AUC	0.622			0.680			0.706		

Abbreviations: AUC, area under the curve; CI, confidence interval; GS, glutamine synthetase.

### 3.3 Prediction Results for IHC Marker CD10

**FIGURE 3:**
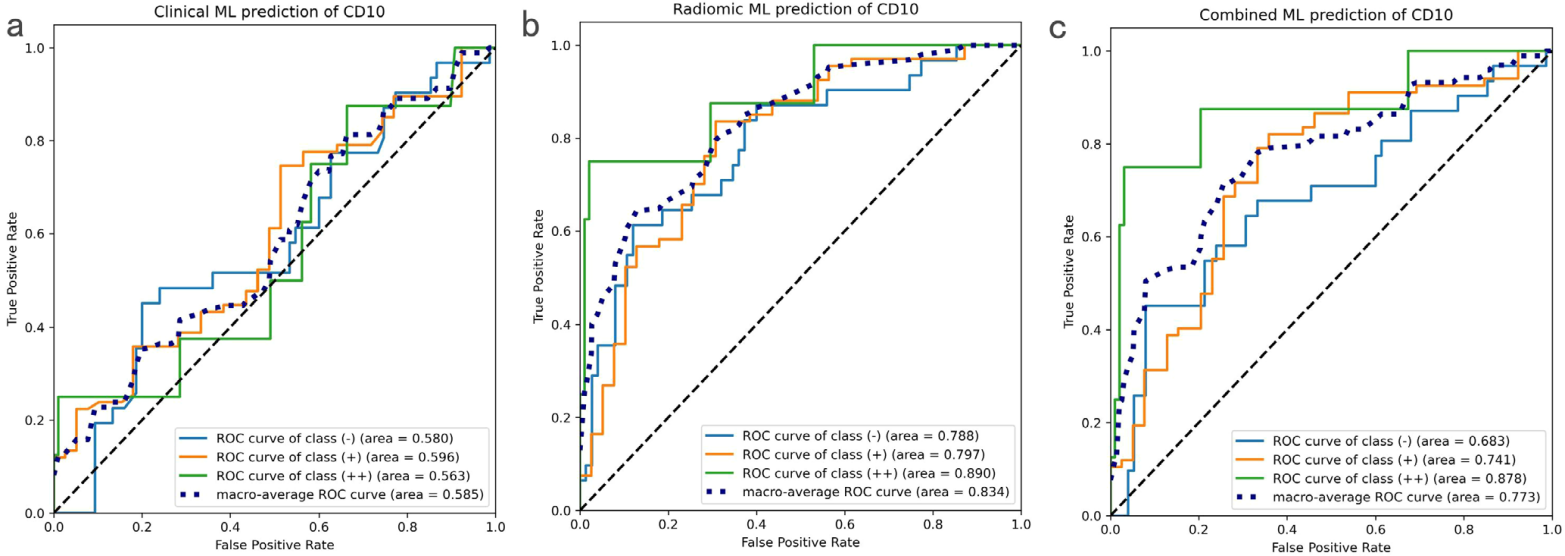
ROC curves for clinical, radiomics, and combined machine learning prediction of CD10. ROC curves for the clinical (clinical biomarkers TBil and DBil) classifier (a), radiomics classifier (b), and the combined clinical‐radiomics classifier (c) for the multiclass prediction of CD10. The macro‐averaged AUC of the radiomics classifier was 0.834.

For CD10 expression, 14 radiomic features from B‐mode images and 4 features from the Kupffer phase were selected using the ANOVA–Spearman process (Table 4) (Table S2, Table S5). SHAP summary bar plot (Figure S2) ranks the mean absolute SHAP values for all 18 features. The top‐ranking features were log‐sigma‐0‐1‐mm‐3D_firstorder_90Percentile (BM, SHAP value = 0.261), log‐sigma‐0‐2‐mm‐3D_glcm_ClusterProminence (BM, SHAP value = 0.237), and lbp‐2D_firstorder_10Percentile (KP, SHAP value = 0.182). The lowest‐ranking feature was original_glcm_DifferenceEntropy (BM, SHAP value = 0.047). ROC analysis revealed that the clinical classifier, based on TBil and DBil, performed less effectively compared to the radiomics classifier. The radiomics classifier demonstrated superior discrimination performance, with the highest macro‐averaged AUC of 0.834, significantly outperforming the clinical approach. The combined classifier showed improved performance across all metrics (Figure 3, Table 5), with the NRI for the addition of radiomic features to TBil/DBil being 0.137, indicating a positive impact on classification accuracy.

**TABLE 4:** ANOVA‐Spearman selected radiomics features for CD10 expression classification.

B‐mode	Kupffer
log‐sigma‐0‐1‐mm‐3D_firstorder_90Percentile	log‐sigma‐0‐1‐mm‐3D_glszm_SizeZoneNonUniformity
log‐sigma‐0‐2‐mm‐3D_firstorder_Maximum	log‐sigma‐0‐1‐mm‐3D_glszm_SizeZoneNonUniformityNormalized
log‐sigma‐0‐4‐mm‐3D_glcm_MaximumProbability	wavelet‐HH_gldm_DependenceNonUniformityNormalized
log‐sigma‐0‐4‐mm‐3D_ngtdm_Strength	wavelet‐LL_gldm_LargeDependenceLowGrayLevelEmphasis
log‐sigma‐0‐5‐mm‐3D_glcm_InverseVariance	
log‐sigma‐0‐5‐mm‐3D_ngtdm_Contrast	
original_glcm_Contrast	
wavelet‐LH_firstorder_10Percentile	
wavelet‐LH_glcm_Correlation	
wavelet‐HL_gldm_DependenceVariance	
wavelet‐HH_firstorder_Kurtosis	
wavelet‐HH_firstorder_Median	
wavelet‐HH_glcm_Imc1	
wavelet‐HH_glcm_Imc2	

Abbreviation: ANOVA, analysis of variance.

**TABLE 5:** Diagnostic summary for CD10 classifiers.

	Clinical classifier			Radiomic classifier			Joint classifier		
	(−)	(+)	(++)	(−)	(+)	(++)	(−)	(+)	(++)
(−)	0	30	1	15	16	1	14	16	1
(+)	4	63	0	5	60	2	7	59	1
(++)	0	6	2	1	1	6	1	5	2
Accuracy	0.609 (95% CI: 0.605, 0.614)			0.766 (95% CI: 0.762, 0.769)			0.709 (95% CI: 0.705, 0.713)		
F1 score	0.362 (95% CI: 0.356, 0.368)			0.704 (95% CI: 0.699, 0.710)			0.534 (95% CI: 0.528, 0.541)		
Cohen's *κ*	0.024 (95% CI: 0.018, 0.029)			0.503 (95% CI: 0.495, 0.511)			0.357 (95% CI: 0.350, 0.365)		
Per‐class AUC	0.580	0.596	0.563	0.788	0.797	0.890	0.683	0.741	0.878
Macro‐averaged AUC	0.585			0.834			0.773		

Abbreviations: AUC, area under the curve; CI, confidence interval.

### 3.4 Prediction Result for the IHC Marker GPC3

**FIGURE 4:**
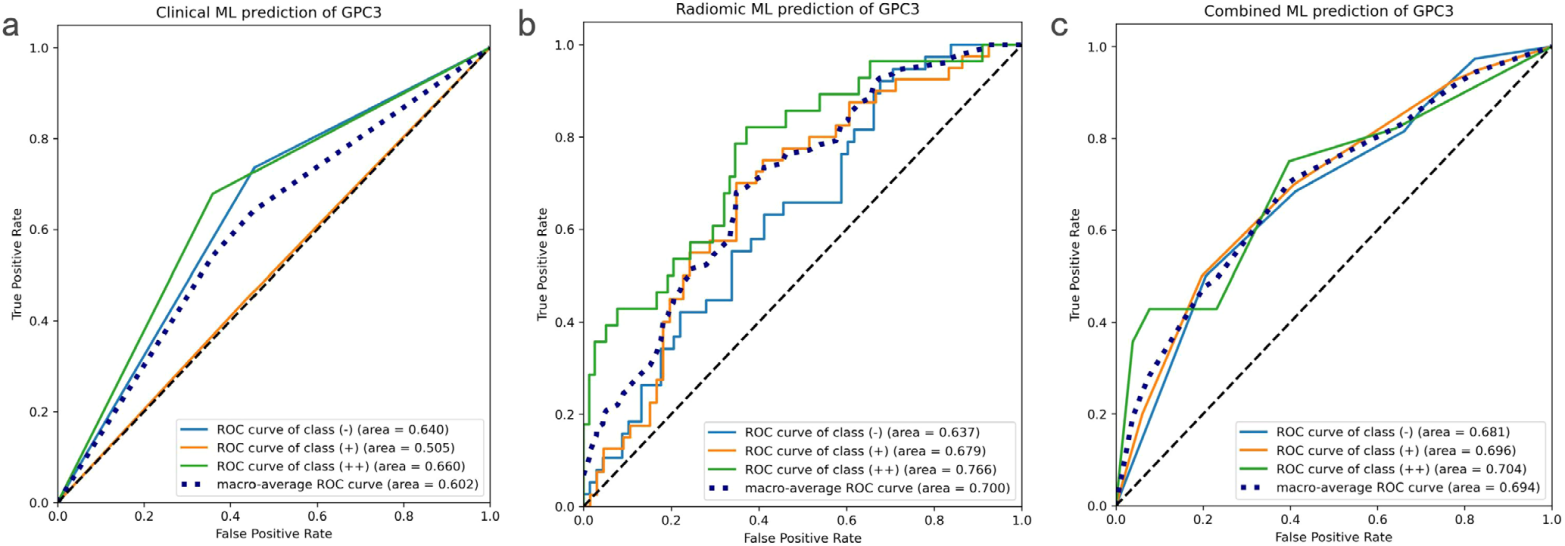
ROC curves for clinical, radiomics, and combined machine learning prediction of GPC3. ROC curves for the clinical (pathological grade) classifier (a), radiomics classifier (b), and the combined clinical‐radiomics classifier (c) for the multiclass prediction of GPC3. The radiomics classifier achieved the highest macro‐averaged AUC of 0.700.

For GPC3 expression, 8 B‐mode radiomic features and 6 Kupffer phase features were selected for analysis (Table 6) (Table S3, Table S5). The top‐ranking features included log‐sigma‐0‐2‐mm‐3D_glrlm_LongRunHighGrayLevelEmphasis (BM, SHAP value = 0.203), wavelet‐HH_glcm_ClusterTendency (BM, SHAP value = 0.184), and lbp‐2D_firstorder_MeanAbsoluteDeviation (KP, SHAP value = 0.149). The lowest‐ranking feature was lbp‐2D_firstorder_Entropy (KP, SHAP value = 0.053) (Figure S3). The radiomics classifier achieved the highest macro‐averaged AUC of 0.700, closely followed by the clinical classifier (AUC = 0.640). The radiomics classifier was more effective at distinguishing positive patients from negative patients, especially when classifying mild and strong expressions. Although the combined classifier did not show improvement in AUC over the radiomics classifier, it still outperformed the clinical classifier alone (Figure 4). The NRI for the addition of radiomic features to the clinical classifier was 0.134, indicating that the combined approach improved clinical predictions, although there was a slight negative NRI (−0.007) when comparing it to the radiomics classifier (Table 7).

**TABLE 6:** ANOVA‐Spearman selected radiomics features for GPC3 expression classification.

B‐mode	Kupffer
log‐sigma‐0‐2‐mm‐3D_glrlm_LongRunHighGrayLevelEmphasis	original_shape2D_Elongation
log‐sigma‐0‐4‐mm‐3D_glrlm_LongRunHighGrayLevelEmphasis	log‐sigma‐0‐4‐mm‐3D_glcm_Autocorrelation
log‐sigma‐0‐4‐mm‐3D_ngtdm_Contrast	original_glcm_Imc2
log‐sigma‐0‐5‐mm‐3D_glcm_Autocorrelation	wavelet‐HH_gldm_DependenceNonUniformityNormalized
original_glszm_LargeAreaHighGrayLevelEmphasis	wavelet‐HH_gldm_DependenceVariance
wavelet‐HL_firstorder_Median	wavelet‐LL_glcm_MCC
wavelet‐HH_glcm_Imc1	
wavelet‐HH_glszm_SizeZoneNonUniformityNormalized	

Abbreviations: ANOVA, analysis of variance; GPC3, glypican‐3.

**TABLE 7:** Diagnostic summary for GPC3 classifiers.

	Clinical classifier			Radiomic classifier			Joint classifier		
	(−)	(+)	(++)	(−)	(+)	(++)	(−)	(+)	(++)
(−)	28	0	10	24	11	3	26	9	3
(+)	22	0	18	17	20	3	21	16	3
(++)	9	0	19	14	2	12	7	9	12
Accuracy	0.441 (95% CI: 0.437, 0.446)			0.526 (95% CI: 0.522, 0.530)			0.511 (95% CI: 0.507, 0.516)		
F1 score	0.357 (95% CI: 0.354, 0.360)			0.519 (95% CI: 0.515, 0.524)			0.500 (95% CI: 0.495, 0.504)		
Cohen's *κ*	0.180 (95% CI: 0.176, 0.185)			0.272 (95% CI: 0.265, 0.278)			0.244 (95% CI: 0.238, 0.250)		
Per‐class AUC	0.640	0.505	0.660	0.637	0.679	0.766	0.681	0.696	0.704
Macro‐averaged AUC	0.602			0.700			0.694		

Abbreviations: AUC, area under the curve; CI, confidence interval.

### 3.5 Prediction Result for the IHC Marker HSP70

**FIGURE 5:**
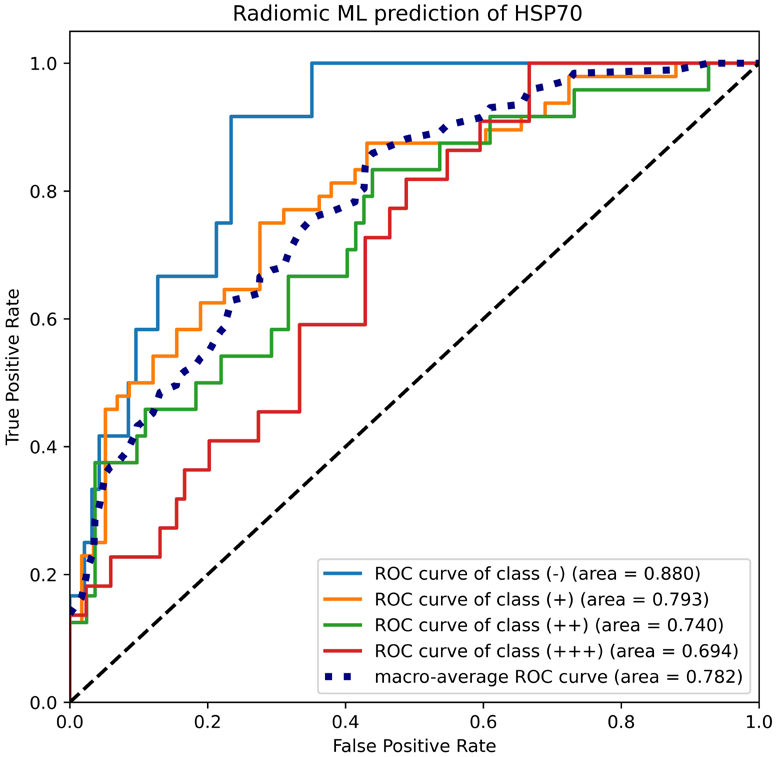
ROC curves for radiomic machine learning prediction of HSP70. The radiomic classifier demonstrated excellent performance in identifying negative and positive HSP70 expression (AUC = 0.880).

For HSP70 expression, 14 radiomic features (7 from each ultrasound mode) were identified through the ANOVA–Spearman process (Table 8) (Table S4, Table S5). The Naïve Bayes classifier, based on these radiomic features, demonstrated excellent performance in identifying negative and positive HSP70 expression (AUC = 0.880) (Figure 5). However, its performance was less robust in distinguishing strongly positive (+++) from other expression levels, with an AUC of only 0.694. These results highlight that while the classifier was effective for lower expression levels, it faced challenges in categorizing the highest expression group (Table 9).

**TABLE 8:** ANOVA‐Spearman selected radiomics features for HSP70 expression classification.

B‐mode	Kupffer
original_ngtdm_Busyness	wavelet‐LL_glcm_Imc1
wavelet‐HH_glszm_SmallAreaLowGrayLevelEmphasis	lbp‐2D_gldm_DependenceEntropy
original_glcm_Correlation	wavelet‐HH_glcm_Idmn
log‐sigma‐0‐2‐mm‐3D_firstorder_10Percentile	log‐sigma‐0‐4‐mm‐3D_glszm_LowGrayLevelZoneEmphasis
log‐sigma‐0‐1‐mm‐3D_firstorder_Uniformity	wavelet‐HL_glcm_Idmn
original_glcm_Imc1	lbp‐2D_gldm_DependenceNonUniformityNormalized
wavelet‐HL_firstorder_Median	wavelet‐HL_gldm_SmallDependenceLowGrayLevelEmphasis

Abbreviations: ANOVA, analysis of variance; HSP, heat shock protein.

**TABLE 9:** Diagnostic summary for HSP70 classifier.

	Radiomics classifier			
	(−)	(+)	(++)	(+++)
(−)	11	1	0	0
(+)	13	31	0	4
(++)	10	10	3	1
(+++)	9	7	2	4
Accuracy	0.459 (95% CI: 0.454, 0.463)			
F1 score	0.365 (95% CI: 0.361, 0.369)			
Cohen's *κ*	0.243 (95% CI: 0.239, 0.248)			
Per‐class AUC	0.880	0.793	0.740	0.694
Macro‐averaged AUC	0.782			

Abbreviations: AUC, area under the curve; CI, confidence interval; HSP, heat shock protein.

## 4 Discussion

With the development of precision medicine, identifying new quantitative and radiomics‐based noninvasive imaging biomarkers aimed at improving the predictive performance of medical images has become an area of interest in radiological research [19, 20, 21, 22]. While radiomics has emerged as a powerful tool for extracting high‐dimensional features from medical images, existing research has largely focused on conventional imaging modalities, such as CT and MRI, and on invasive biopsy methods for biomarker prediction. A previous study showed that the nomogram based on LI‐RADS features, MRI, and clinical indicators could accurately predict GPC‐3 expression in HCC [23]. Despite these advances, there remains a significant gap in the noninvasive prediction of IHC biomarkers using ultrasound‐based radiomics, particularly with regard to the Kupffer phase of S‐CEUS images. The research gap addressed by this study lies in the novel application of radiomics features extracted from the Kupffer phase of S‐CEUS, which provides unique insights into the microvascular environment of HCC. This noninvasive imaging technique has the potential to predict key IHC markers, such as GPC3, HSP70, GS, and CD10, which are traditionally assessed through biopsy or surgery [24, 25]. Previous studies have shown the utility of radiomics for predicting Ki‐67 expression using the Kupffer phase of S‐CEUS images, but to our knowledge, no other studies have integrated the quantitative analysis of clinical factors with radiomic features for the prediction of IHC biomarkers in HCC [13, 17]. HCC is one of the most common cancers worldwide, closely associated with tumor‐associated macrophages (TAMs), including monocyte‐derived macrophages and Kupffer cells [26]. The M2 phenotype of macrophages particularly promotes the progression of HCC and is associated with poor prognosis in HCC patients. GPC‐3 expression reduces the adhesion of HCC cells to the extracellular matrix and stimulates cell migration and invasion, which might promote HCC metastasis. The expression of GPC‐3 on the cell membrane has been shown to reactivate the recruitment of macrophages, which is different from typical Kupffer cells and a trend of migrating macrophages morphology [27]. However, the connection between migrating macrophages and TAMs' Sonazoid uptake inhibition remains to be explored [28].

Our study addresses this gap by using radiomic features from both the B‐mode and Kupffer phases of S‐CEUS to predict IHC markers that are usually obtained through invasive methods. The data indicate that the radiomics model derived from Kupffer phase S‐CEUS images showed promising results in predicting biomarkers, such as GPC3 and CD10, suggesting its potential clinical applicability. This approach is particularly significant because it opens up the possibility of noninvasive monitoring of HCC biomarkers, which is highly beneficial for patient care, especially in cases where biopsy is not feasible. In addition, the combination of clinical risk factors with radiomic features provided a more effective classifier for biomarkers, such as GS and GPC3 expression than either clinical or radiomic features alone; although it did not improve the AUC results compared to the radiomics‐only model. This highlights the importance of radiomics as a robust predictor in isolation while also showing that combining clinical factors can enhance predictive accuracy for certain biomarkers. The clinical significance of these findings lies in the potential to reduce the need for invasive biopsies for IHC biomarker assessment, enabling noninvasive monitoring of HCC progression and guiding personalized treatment decisions, such as selecting targeted therapies based on GS or GPC3 expression. However, the clinical utility requires further validation in larger, multicenter cohorts to confirm applicability in routine practice.

Radiomic features, including texture, intensity, and shape, can capture the complex heterogeneity of malignant lesions that might be missed by conventional imaging or subjective interpretation by radiologists [29]. Our study's findings are consistent with the emerging trend in precision medicine, where radiomics is being used to decode the relationship between imaging characteristics and underlying tumor biology [30]. Furthermore, the high‐throughput predictive capabilities of radiomics, such as tumor surrounding dilation, offer a comprehensive “virtual tissue pathology” approach that quantifies tissue properties at a microscopic level, presenting a more detailed understanding of the tumor microenvironment [30]. In terms of the limitations, our study is limited by its single‐center design and relatively small sample size (*n* = 86 patients, 106 nodules), which may contribute to class imbalance (e.g., 7.5% CD10++ cases) and potentially overoptimistic AUCs. Additionally, image quality control was limited to manual checks by radiologists for artifacts and noise, without standardized protocols for ultrasound imaging settings (e.g., gain, depth), which may introduce variability in radiomic feature extraction. Data bias is another limitation, as the single‐center cohort may not represent diverse patient populations, potentially leading to selection bias and reduced generalizability. The ANOVA‐Spearman feature selection method, while effective for reducing dimensionality, may overlook nonlinear relationships or interactions between features, limiting the capture of complex predictive patterns. These limitations could affect the robustness of the Naïve Bayes classifiers, particularly for markers with lower AUCs like GPC3 (0.694, Table 7). To address these limitations in future work, we plan to implement automated image quality control protocols to standardize ultrasound imaging parameters, reducing feature variability. Multicenter cohorts with broader demographic representation will be pursued to mitigate data bias and enhance generalizability. Advanced feature selection methods, such as machine learning‐based approaches (e.g., recursive feature elimination, mutual information), will be explored to capture nonlinear relationships and improve model performance. Multi‐fold cross‐validation (e.g., 5‐fold) or external validation cohorts will also be incorporated to provide more robust performance estimates. The radiomic features from the arterial phase were found to be less effective, leading to their exclusion from the analysis. Future investigations should focus on expanding the diagnostic value by including dynamic graph features from multiple phases of S‐CEUS, including the arterial, portal venous, late phase, and Kupffer phase. Additionally, the lack of multimodal radiological data in our study is another limitation. The integration of S‐CEUS with other imaging modalities, such as gadoxetic acid‐enhanced MRI and CT, could further enhance predictive accuracy and provide a more holistic approach to biomarker prediction. Future work should explore the potential of combining ultrasound‐based radiomics with MRI and CT data to refine and improve the predictive performance of IHC biomarkers in HCC. Although supplementary mixed‐effects modeling suggested minimal clustering effects, intra‐patient lesion correlation warrants further investigation in larger cohorts.

## 5 Conclusions

In conclusion, the Kupffer phase in the S‐CEUS‐based radiomics signature is an excellent biomarker. It has achieved desirable results in predicting the IHC expression of GPC3, GS, HSP70, and CD10 in patients with HCC. The combined clinical factors and radiomics signature may provide an effective tool for the noninvasive and individualized prediction of immunohistochemistry expression in HCC. Future work will focus on validating these results in multicenter cohorts to enhance clinical applicability.

## Author Contributions

**Chen Li:** conceptualization, data curation, methodology, project administration, writing – original draft, writing – review and editing. **Yuan Liu:** data curation, formal analysis, software, writing – review and editing. **Mingxiao Wu:** supervision, investigation, funding acquisition. **Weide Dai:** funding acquisition, visualization, supervision. **Jinghai Song:** supervision, resources. **Yong Wang:** supervision, funding acquisition, project administration, writing – review and editing.

## Ethics Statement

The study was approved by the Ethics Committees of Beijing Hospital (ethics approval letter no. 2022BJYYEC‐029‐02).

## Conflicts of Interest

The authors declare no conflicts of interest.

## Supporting information


**Figure S1:** SHAP summary bar plot for GS expression (Naïve Bayes). The SHAP summary bar plot ranks the mean absolute SHAP values for all 23 features.

**Figure S2:** SHAP summary bar plot for CD10 expression (Naïve Bayes). The SHAP summary bar plot ranks the mean absolute SHAP values for all 18 features.

**Figure S3:** SHAP summary bar plot for GPC3 expression (Naïve Bayes). SHAP summary bar plot ranks the mean absolute SHAP values for all 14 features.

**Figure S4:** SHAP summary bar plot for HSP70 expression (Naïve Bayes). SHAP summary bar plot ranks the mean absolute SHAP values for all 14 features.

**Table S1:** The mixed effects regression summary for GS.

**Table S2:** The mixed effects regression summary table for CD10.

**Table S3:** The mixed effects regression summary for GPC3.

**Table S4:** The mixed effect regression summary for HSP70.

**Table S5:** The mixed effects performance metric for IHC markers.

## Data Availability

All data generated or analyzed during this study are included in this article and Supporting Information.
