# Supplementary figures and images for: Kupffer Phase Radiomics Signature in Sonazoid Contrast‐Enhanced Ultrasound Predicts Immunohistochemistry Marker Expression in Hepatocellular Carcinoma
Source: Cancer Med. 2025 Oct 6;14(19):e71153. doi: 10.1002/cam4.71153 (PMC12497941; doi:10.1002/cam4.71153)

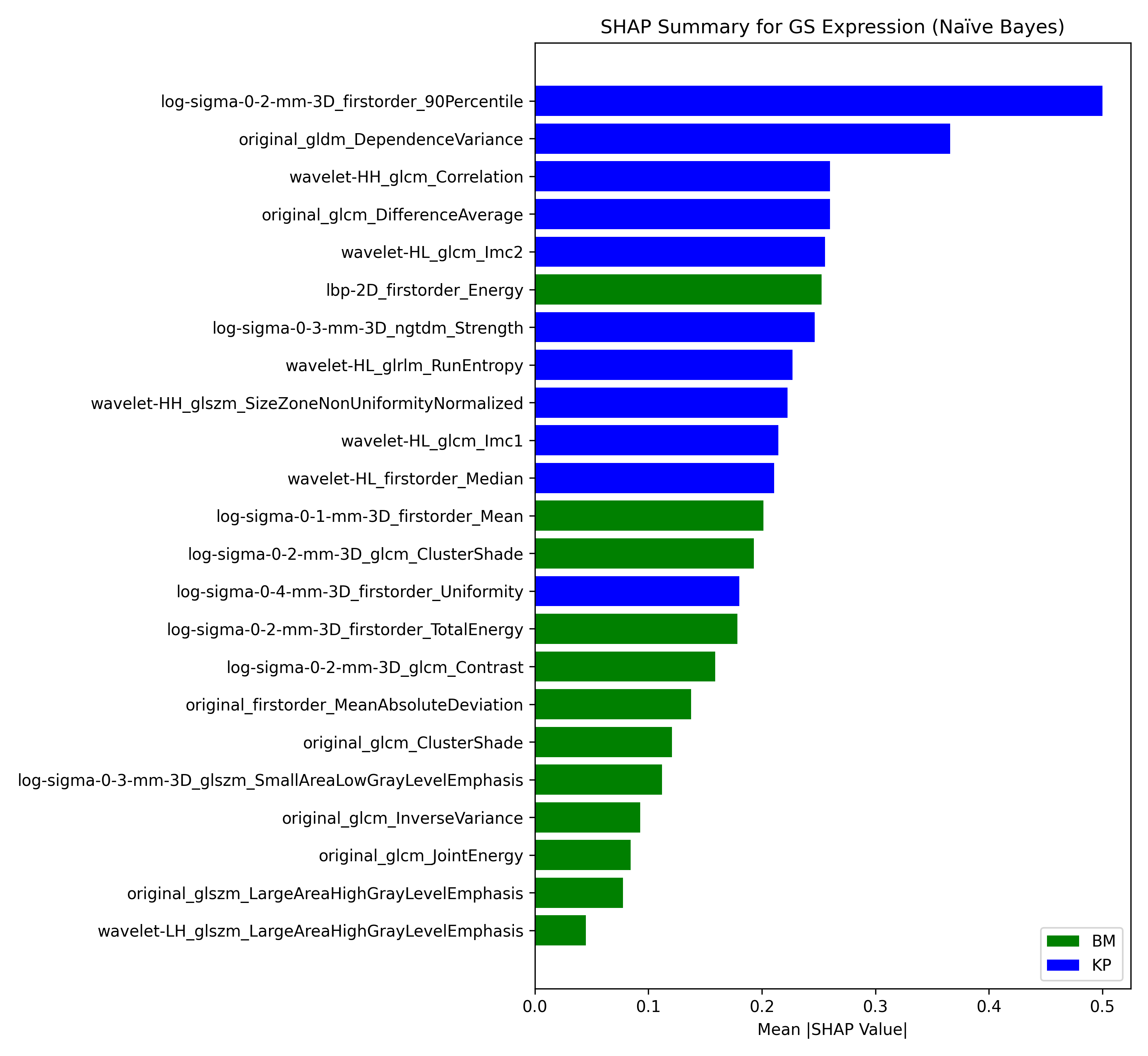

Supplement: Supplementary file 1 — Figure S1: SHAP summary bar plot for GS expression (Naïve Bayes). The SHAP summary bar plot ranks the mean absolute SHAP values for all 23 features. [file CAM4-14-e71153-s002.png]

SHAP Summary for CD10 Expression (Naïve Bayes)

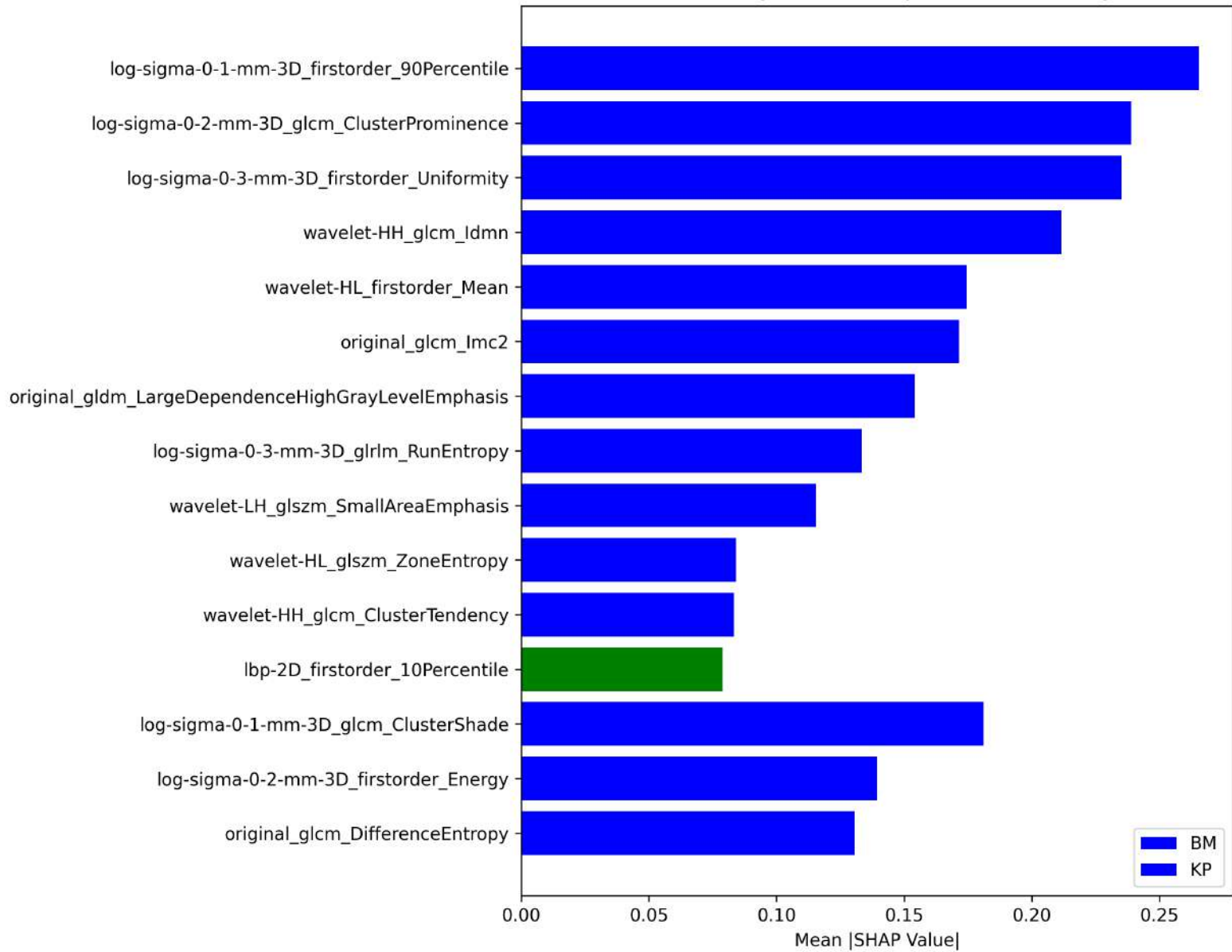

Supplement: Supplementary file 2 — Figure S2: SHAP summary bar plot for CD10 expression (Naïve Bayes). The SHAP summary bar plot ranks the mean absolute SHAP values for all 18 features. [file CAM4-14-e71153-s009.pdf]

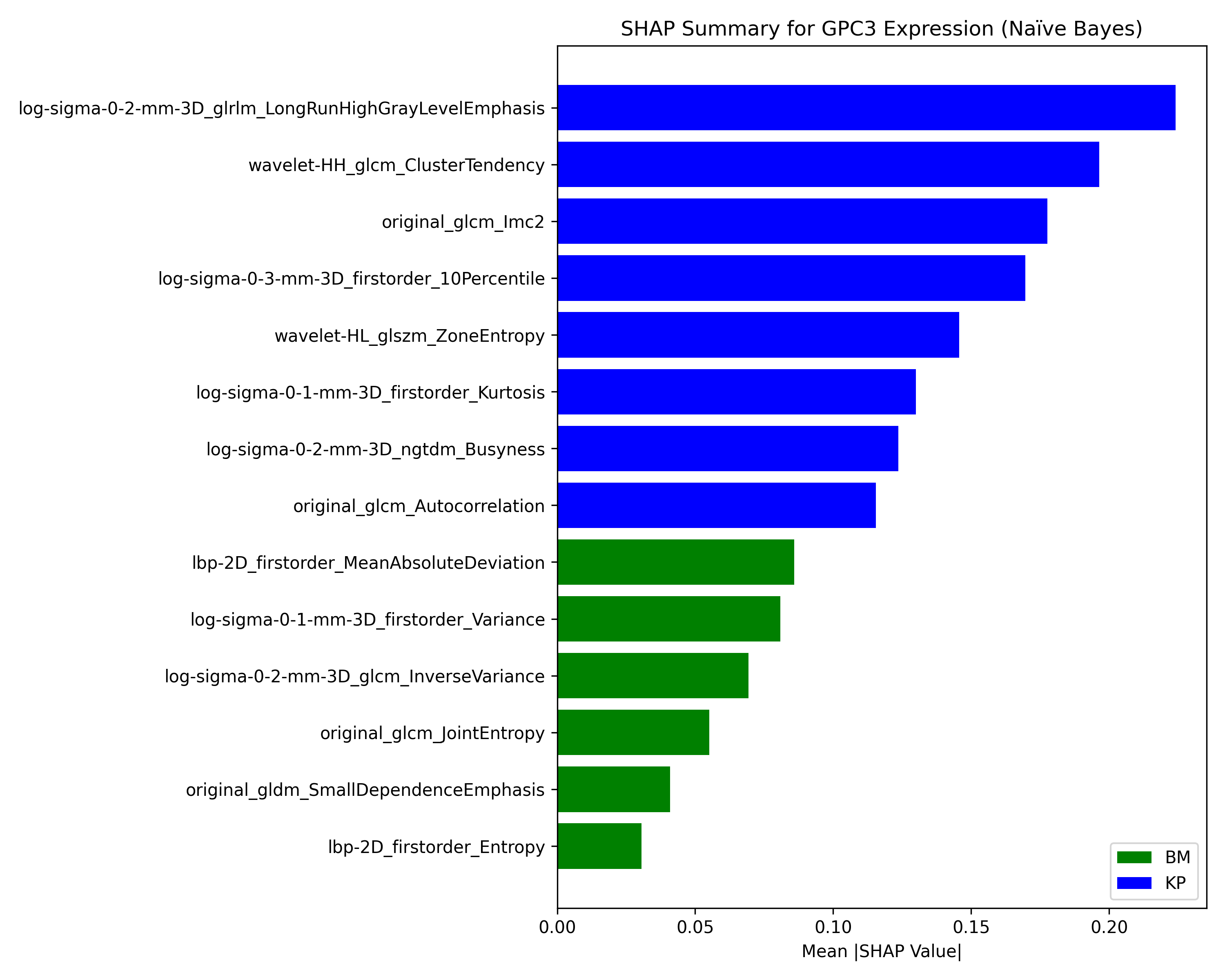

Supplement: Supplementary file 3 — Figure S3: SHAP summary bar plot for GPC3 expression (Naïve Bayes). SHAP summary bar plot ranks the mean absolute SHAP values for all 14 features. [file CAM4-14-e71153-s004.png]

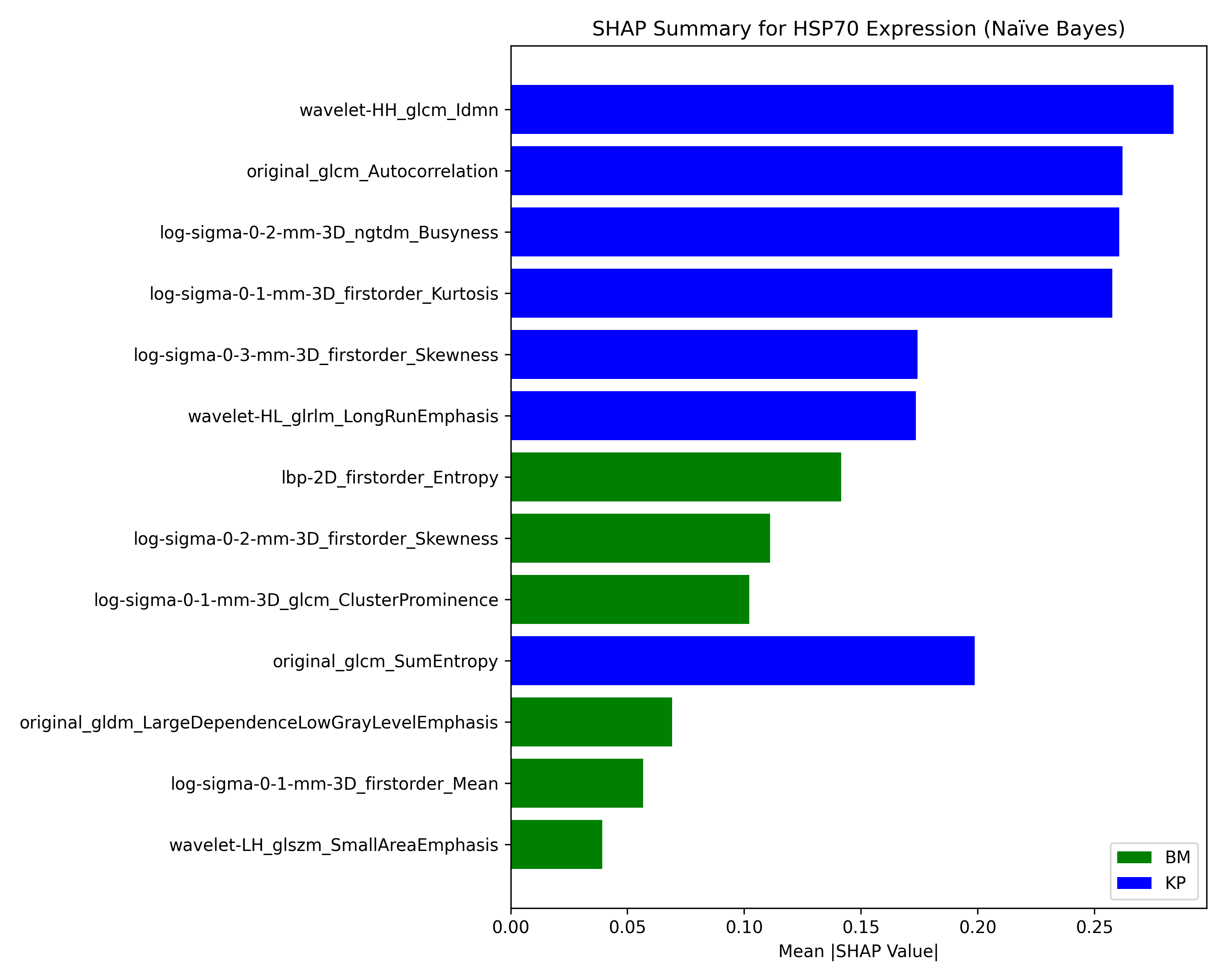

Supplement: Supplementary file 4 — Figure S4: SHAP summary bar plot for HSP70 expression (Naïve Bayes). SHAP summary bar plot ranks the mean absolute SHAP values for all 14 features. [file CAM4-14-e71153-s003.png]
