# Supplementary material for: Kupffer Phase Radiomics Signature in Sonazoid Contrast‐Enhanced Ultrasound Predicts Immunohistochemistry Marker Expression in Hepatocellular Carcinoma
Source: Cancer Med. 2025 Oct 6;14(19):e71153. doi: 10.1002/cam4.71153 (PMC12497941; doi:10.1002/cam4.71153)
Supplement: Supplementary file 5 — Table S1: The mixed effects regression summary for GS. [file CAM4-14-e71153-s008.docx]

| Variable | Coef | Std.Err | z | P>\|z\| | [0.025 | 0.975] |
| --- | --- | --- | --- | --- | --- | --- |
| Intercept | -0.15 | 0.12 | -1.25 | 0.211 | -0.385 | 0.085 |
| log-sigma-0-2-mm-3D_firstorder_90Percentile | 0.32 | 0.095 | 3.368 | 0.001 | 0.134 | 0.506 |
| log-sigma-0-3-mm-3D_ngtdm_Strength | 0.28 | 0.09 | 3.111 | 0.002 | 0.103 | 0.457 |
| wavelet-HL_glcm_Imc1 | 0.25 | 0.085 | 2.941 | 0.003 | 0.083 | 0.417 |
| log-sigma-0-2-mm-3D_glcm_ClusterShade | -0.22 | 0.08 | -2.75 | 0.006 | -0.377 | -0.063 |
| original_glcm_JointEnergy | 0.2 | 0.075 | 2.667 | 0.008 | 0.053 | 0.347 |
| Random Effect: patient_id (Variance) | 0.45 |  |  |  |  |  |
| Random Effect: patient_id (Std.Dev) | 0.671 |  |  |  |  |  |

Table S1 The mixed effects regression summary for GS
