# Supplementary material for: Kupffer Phase Radiomics Signature in Sonazoid Contrast‐Enhanced Ultrasound Predicts Immunohistochemistry Marker Expression in Hepatocellular Carcinoma
Source: Cancer Med. 2025 Oct 6;14(19):e71153. doi: 10.1002/cam4.71153 (PMC12497941; doi:10.1002/cam4.71153)
Supplement: Supplementary file 6 — Table S2: The mixed effects regression summary table for CD10. [file CAM4-14-e71153-s001.docx]

| Variable | Coef | Std.Err | z | P>\|z\| | [0.025 | 0.975] |
| --- | --- | --- | --- | --- | --- | --- |
| Intercept | -0.2 | 0.13 | -1.538 | 0.124 | -0.455 | 0.055 |
| log-sigma-0-1-mm-3D_firstorder_90Percentile | 0.29 | 0.1 | 2.9 | 0.004 | 0.094 | 0.486 |
| log-sigma-0-2-mm-3D_glcm_ClusterProminence | -0.26 | 0.095 | -2.737 | 0.006 | -0.446 | -0.074 |
| wavelet-HH_glcm_Idmn | 0.23 | 0.09 | 2.556 | 0.011 | 0.053 | 0.407 |
| lbp-2D_firstorder_10Percentile | 0.21 | 0.085 | 2.471 | 0.013 | 0.043 | 0.377 |
| original_glcm_DifferenceEntropy | -0.19 | 0.08 | -2.375 | 0.018 | -0.347 | -0.033 |
| Random Effect: patient_id (Variance) | 0.42 |  |  |  |  |  |
| Random Effect: patient_id (Std.Dev) | 0.648 |  |  |  |  |  |

Table S2 The mixed effects regression summary table for CD10
