# Supplementary material for: Kupffer Phase Radiomics Signature in Sonazoid Contrast‐Enhanced Ultrasound Predicts Immunohistochemistry Marker Expression in Hepatocellular Carcinoma
Source: Cancer Med. 2025 Oct 6;14(19):e71153. doi: 10.1002/cam4.71153 (PMC12497941; doi:10.1002/cam4.71153)
Supplement: Supplementary file 7 — Table S3: The mixed effects regression summary for GPC3. [file CAM4-14-e71153-s006.docx]

| Variable | Coef | Std.Err | z | P>\|z\| | [0.025 | 0.975] |
| --- | --- | --- | --- | --- | --- | --- |
| Intercept | -0.25 | 0.14 | -1.786 | 0.074 | -0.524 | 0.024 |
| log-sigma-0-2-mm-3D_glrlm_LongRunHighGrayLevelEmphasis | 0.27 | 0.105 | 2.571 | 0.01 | 0.064 | 0.476 |
| original_glcm_Imc2 | -0.24 | 0.1 | -2.4 | 0.016 | -0.436 | -0.044 |
| wavelet-HL_glszm_ZoneEntropy | 0.22 | 0.095 | 2.316 | 0.021 | 0.034 | 0.406 |
| lbp-2D_firstorder_MeanAbsoluteDeviation | 0.2 | 0.09 | 2.222 | 0.026 | 0.023 | 0.377 |
| original_gldm_SmallDependenceEmphasis | -0.18 | 0.085 | -2.118 | 0.034 | -0.347 | -0.013 |
| Random Effect: patient_id (Variance) | 0.4 |  |  |  |  |  |
| Random Effect: patient_id (Std.Dev) | 0.632 |  |  |  |  |  |

Table S3 The mixed effects regression summary for GPC3
