# Supplementary material for: Kupffer Phase Radiomics Signature in Sonazoid Contrast‐Enhanced Ultrasound Predicts Immunohistochemistry Marker Expression in Hepatocellular Carcinoma
Source: Cancer Med. 2025 Oct 6;14(19):e71153. doi: 10.1002/cam4.71153 (PMC12497941; doi:10.1002/cam4.71153)
Supplement: Supplementary file 8 — Table S4: The mixed effect regression summary for HSP70. [file CAM4-14-e71153-s005.docx]

| Variable | Coef | Std.Err | z | P>\|z\| | [0.025 | 0.975] |
| --- | --- | --- | --- | --- | --- | --- |
| Intercept | -0.3 | 0.15 | -2 | 0.046 | -0.594 | -0.006 |
| log-sigma-0-1-mm-3D_firstorder_Kurtosis | 0.26 | 0.11 | 2.364 | 0.018 | 0.044 | 0.476 |
| wavelet-HH_glcm_Idmn | 0.24 | 0.105 | 2.286 | 0.022 | 0.034 | 0.446 |
| lbp-2D_firstorder_Entropy | -0.22 | 0.1 | -2.2 | 0.028 | -0.416 | -0.024 |
| log-sigma-0-2-mm-3D_firstorder_Skewness | 0.2 | 0.095 | 2.105 | 0.035 | 0.014 | 0.386 |
| original_glcm_SumEntropy | -0.18 | 0.09 | -2 | 0.046 | -0.357 | -0.003 |
| Random Effect: patient_id (Variance) | 0.43 |  |  |  |  |  |
| Random Effect: patient_id (Std.Dev) | 0.656 |  |  |  |  |  |

Table S4 The mixed effect regression summary for HSP70
