# Supplementary material for: Kupffer Phase Radiomics Signature in Sonazoid Contrast‐Enhanced Ultrasound Predicts Immunohistochemistry Marker Expression in Hepatocellular Carcinoma
Source: Cancer Med. 2025 Oct 6;14(19):e71153. doi: 10.1002/cam4.71153 (PMC12497941; doi:10.1002/cam4.71153)
Supplement: Supplementary file 9 — Table S5: The mixed effects performance metric for IHC markers. [file CAM4-14-e71153-s007.docx]

Table S5 The mixed effects performance metric for IHC markers

| IHC markers | Metric | Mean | CI_Lower | CI_Upper |
| --- | --- | --- | --- | --- |
| GS | AUC | 0.912 | 0.89 | 0.934 |
|  | Accuracy | 0.538 | 0.51 | 0.566 |
|  | F1 Score | 0.464 | 0.435 | 0.493 |
| CD10 | AUC | 0.733 | 0.89 | 0.934 |
|  | Accuracy | 0.739 | 0.71 | 0.768 |
|  | F1 Score | 0.564 | 0.534 | 0.594 |
| GPC3 | AUC | 0.644 | 0.618 | 0.67 |
|  | Accuracy | 0.451 | 0.422 | 0.48 |
|  | F1 Score | 0.44 | 0.41 | 0.47 |
| HSP70 | AUC | 0.822 | 0.798 | 0.846 |
|  | Accuracy | 0.519 | 0.489 | 0.549 |
|  | F1 Score | 0.325 | 0.295 | 0.355 |
